# Butterflies, Dwarves, and Plastic Lollypops: A Case Report on Medical Clowning in a Children’s Rehabilitation Hospital

**DOI:** 10.3390/children9121805

**Published:** 2022-11-24

**Authors:** Sigalit Ofer, Shoshi Keisari

**Affiliations:** 1 Alyn Hospital, Pediatric and Adolescent Rehabilitation Center, Jerusalem 91090, Israel; 2 The Dream Doctor Project, Medical Clowning in Action, Tel Aviv 6701751, Israel; 3 The Drama Therapy Program, Tel-Hai College, Upper Galilee, 9977, Qiryat Shemona 1220800, Israel; 4 Drama & Health Science Lab, The School of Creative Arts Therapies, Faculty of Social Welfare and Health Sciences, University of Haifa, Mount Carmel, Haifa 3498838, Israel; 5 The Emili Sagol Creative Arts Therapies Research Center, Faculty of Social Welfare and Health Sciences, University of Haifa, Mount Carmel, Haifa 3498838, Israel

**Keywords:** rehabilitation, medical clowning, drama therapy, physiotherapy, role theory

## Abstract

Medical clowning has been evolving in the past three decades and now plays a significant role in the rehabilitation processes of children who have suffered injuries and undergone complex medical procedures. The current paper focuses on this topic by presenting a case study of a young girl who lost most of her functional abilities due to brain damage. During the child’s physiotherapy sessions at the rehabilitation hospital, a medical clown was brought in to work together with the physiotherapist in providing the treatment. The case study brings an in-depth perspective on the therapeutic process, as it is based on documentation of the sessions while addressing key stages in the child’s rehabilitation, alongside core concepts in drama therapy. The qualitative analysis shows how the playful space in the rehabilitation process enhanced the child’s inner motivation, provided a space for role expansion, and promoted the connection between the child and the environment. This paper demonstrates how the involvement of medical clowns can promote the rehabilitation processes of children who have suffered traumatic injuries and help them cope with functional losses.

## 1. Introduction

### 1.1. The Role of the Medical Clown in the Hospital

The character of the clown has accompanied humankind since the dawn of civilization, as a spiritual leader, shaman, and healer, and has played a counter role to key roles in society such as the ruler and the priest [1]. The clowns’ humor stems from their ability to embody the gap between the spiritual and corporeal, as well as between the tragic and the comic, thus providing a broad and positive perspective on human existence [2].

The clowns’ most prominent feature is their paradoxical nature. “Whatever predicate we use to describe them, the opposite can also be said, and with equal right” [3] (p. 307). The constant inner contradiction embodied in the character of the clowns constitutes their inner logic and allows them to be flexible in in-between situations, constantly shifting between dichotomies. The clown can serve as a mediator between contradictions, examine the relationship between them, and deliver an unconventional perspective on reality [4]. This quality is particularly meaningful and important in a hospital environment, where people often need to cope with stressful and even life-threatening situations.

The medical clown’s role in a hospital is to contribute to the psychological wellbeing of patients, their families, and the attending staff [5,6,7]. Medical clowns operate within the hospital’s reality and connect with patients by interacting with each in a unique way that is tailored to their specific needs and nature [6]. They rely on their ability to improvise and do so out of a sense of empathy, with an aim to build patients’ resources and empower them [8,9]. Clowns invite patients into an imaginary realm, helping them to momentarily escape the harsh reality of hospitalization. This allows patients to delve into a world in which emotions and situations can be taken to extremes for the purpose of transformation, ultimately providing a fresh perspective on the situation. Studies show how the clown’s working techniques can be conceptualized using drama therapeutic models and theory [2,10].

### 1.2. Key Concepts in Drama Therapy

Drama therapy is a health profession that uses dramatic and theater-based methods with the intention of promoting psychological growth within a therapeutic relationship [11,12]. This section describes how each of the drama therapy core concepts finds expression in the work of medical clowns.

***Dramatic reality*** is a key concept in drama therapy. It is a safe space that integrates reality and fantasy and allows the individual to explore subjective experiences in the present [13]. “It is an *as if* made real, an island of imagination that becomes apparent in the midst of actual life” [13] (p. 272). Its therapeutic power stems from its ability to contain imaginary subjective content, some of which may be unacceptable under normal circumstances, thereby creating a legitimate space for such content within the “real” world [14,15]. Once personal content is projected and explored in the dramatic reality, it undergoes a transformation. This transformation seems to be supported by the fact that something real and concrete has been done with the subjective content. Thus, the dramatic reality is a type of lab in which people can explore emotions and situations and experience potential worlds [13,14].

Medical clowns’ appearance, clothing, red nose, and accessories, create a dramatic reality that surrounds them like a type of aura [2,10]. As a medical clown walks down the hospital hallways, Winnicott’s potential space is with them wherever they go. This is the space in between behavior and contemplation, as well as in between “me” and “not me”—the natural zone where play and creativity thrive [16]. The medical clown embodies this space, inviting patients to play and act in it together with her so they can experience change.

***Aesthetic distancing*** is another key concept in drama therapy and is based in the world of theater [17,18]. The aesthetic experience of theater presents fictional characters and narratives that appear to be removed from the viewers’ personal experience. However, paradoxically, this distancing, along with other aesthetic elements of the experience, allows viewers to draw emotionally closer to the events unfolding on stage, thereby drawing closer to themselves [19]. This is known as aesthetic distancing, and it serves a similar function in drama therapy [17,19]. It holds the balance between the emotional component, which fosters closeness and identification with the dramatic material, and the cognitive component, which allows the individual to reflectively observe what is taking place. Thus, aesthetic distancing makes it possible for individuals to distance themselves from personal content that is too close and painful, as well as reduce its emotional charge, creating a safe space for them to observe and explore their experiences [19,20]. Puppets and masks, for example, are well-known theatrical techniques used in drama therapy to facilitate projective expression and create a distance between the self and the characters presented in the process [21]. Similarly, the clown’s red nose can be seen as a small mask of sorts, signaling the existence of a space of play and highlighting and creating an aesthetic distance for the patients [22].

Medical clowns reflect the emotions and situations that accompany patients’ hospitalization experiences. These include fear, alienation, lack of understanding, helplessness, and pain [8,9]. They do so in an exaggerated and absurd way, so that patients can look at the clown from a safe distance, without the need to associate themselves with these emotions. When a patient identifies with an exaggerated experience that the clown portrays, they will often laugh or smile. For example, when encountering a frightened child, the clown will look for a way to be afraid, perhaps of a small dragon doll, a printed lion on a shirt, the honk of a toy, or anything else they find in the space. The child will laugh at the clown’s reaction and will likely want to frighten the clown over and over again. This allows the child to play with the fear and befriend it from a position of power and a sense of security, as the medical clown copes with the fear projected onto her, which they then perform and process. Therefore, maintaining an aesthetic distance is a significant element of the medical clown’s work. It requires a great deal of sensitivity and the ability to adapt from one moment to the next.

***Dramatic role*** is the third key concept we address in this paper. According to role theory in drama therapy and psychodrama, a role is the actual and tangible form of the self, comprising a set of qualities representing the various facets of the individual [23,24]. Each role has counter roles that often represent denied aspects of the self that the individual or society seeks to avoid. These counter roles contain unexpressed emotions, thoughts, beliefs, and experiences [20]. As the therapeutic process progresses, the role of “the guide” emerges to help integrate conflicting roles. At its core, drama therapy focuses on developing a balanced, flexible, and healthy system of roles, expanding the repertoire of roles available to us, and increasing our flexibility in shifting between them [24].

In the hospitalization process, a person steps into the role of “patient”. They are removed from their daily life, stripped of their identity features, lose their privacy, and become defined by their illness [25]. In terms of role theory, the hospitalization process pushes the patient’s role system out of balance, as the person shrinks into the role of “patient”, which is destabilizing and stress-provoking. By playing in the dramatic reality, the medical clown helps patients shift out of this passive and painful role into more active and pleasurable roles that provide them with a sense of control and connect them to their strengths [2]. The role that clowns play in this regard is vital, especially when coping with traumatic events.

### 1.3. Rehabilitation Following a Traumatic Event

Some children who arrive at a rehabilitation hospital are coping with life-changing traumatic events that have significantly impaired their functioning and put their life in danger [26]. Such events change the life of the child’s entire family and necessitate lengthy rehabilitation and adaptation processes, which at times involve reshaping their personal identity [27]. In some situations, the hospitalization itself, which includes the stages of diagnosis and medical intervention, is accompanied by fear, uncertainty, helplessness, pain, and life-threatening situations that could lead to post-traumatic stress disorder (PTSD) [28].

According to Ben Ari et al. (2018), PTSD in response to hospitalization is highly common among children; however, most of them recover from it naturally. Approximately 25–30% of all children develop chronic post-traumatic symptoms that affect their physical recovery and functioning. Others suffer from significant psychological stress and impaired functioning that continue long after hospitalization as a result of their illness or ongoing painful and invasive medical interventions. Symptoms include hyperarousal, avoidance, and re-experiencing the traumatic event [28]. During rehabilitation, children process the traumatic event and learn to adapt to the new reality; this also involves grieving the loss of abilities and aspects of identity [27].

Stroebe and Schut’s (1999) dual process model of coping with bereavement describes an adaptive process for grief resulting from loss. This process involves coping with two types of stressors, leading to an investment of energy in two opposing coping strategies. The first is **loss-oriented** and involves processing emotions, thoughts, and actions related to loss, such as anger, crying, denial, longing for the past, sadness over lost abilities, and more. The second is **restoration-oriented** and includes coping with the new reality created by the loss. This involves the restructuring of daily life, forming new social relationships, and regrowth. When a grieving individual copes with loss in a healthy way they oscillate between these two strategies, and this creates a healthy adaptation process [29,30,31]. The current study presents a case study of a child who experienced a traumatic event and grieving processes following the loss of functions resulting from brain damage. This case study was designed to examine treatment processes with medical clowns who work with children within a rehabilitative framework, to determine the ways in which dramatic therapeutic work can support the processing of traumatic events and grieving processes in situations of functional loss.

## 2. Materials and Methods

The case study presented here is based on treatments conducted as part of the rehabilitation of a 5 year old girl during physiotherapy sessions. The case study method involves an intensive and focused unit, and it provides access to information that might otherwise be inaccessible via other means of study [32].

Amira was hospitalized in the rehabilitation ward due to brain damage resulting from complications that occurred during a medical procedure (to preserve confidentiality, pseudonyms are used for the girl and physiotherapist). Her treatment at the rehabilitation hospital lasted over 2 years, and, in the current paper, we describe short vignettes from three different periods of the physiotherapy treatment. The physiotherapy sessions were co-conducted by Yael, the physiotherapist, and the medical clown, the first author of this paper, Sigalit Ofer, also known at the rehabilitation hospital by her clown name of Tsumi (“The Attention Seeker”). The analysis is based on treatment records written by the medical clown and an interview with the physiotherapist during which the records for each period were presented to elicit her perspective on the role of the medical clown in the process. The analysis of the records and the interview was designed to better understand the trajectory of the treatment process and the role of the medical clown in its different phases [32]. This study was approved by the Ethics Committee of the University of Haifa (UH approval 403/22).

### 2.1. The Triadic Relationship in the Physiotherapy: The Clown, Physiotherapist, and Child

Children hospitalized in the rehabilitation hospital enter into a treatment routine designed to meet their specific needs. To promote their rehabilitation process, the children are required to be active and cooperative; however, they are often in pain or emotionally overwhelmed by their medical condition. In such situations, moving a sore limb or shifting from one position to another can seem like an impossible task. The tension and uncertainty the children and their parents experience can leave them emotionally overwhelmed, hypervigilant, suspicious, or avoidant. In this context, physiotherapy treatments can cause many children to be resistant and experience great difficulty.

Medical clowns join physiotherapy treatments when the attending staff identifies situations of significant emotional difficulty that hinder rehabilitation processes. Their role is to support the children, reduce their anxiety, increase their sense of control, assist them in coping with the pain, and turn the physiotherapy session into a playful experience in which the children feel safe. At the same time, the clowns also support the physiotherapists and the therapy goals that they define. A medical clown’s involvement in physiotherapy sessions is generally in collaboration and coordination with the physiotherapist.

The role of a medical clown is dual. They stand by the resisting child and support then while simultaneously supporting the goals defined by the physiotherapist. The clown attempts to identify the sources of the child’s resistance and helps the child express this resistance, while simultaneously searching for creative ways to transform it. They seek to expose the child’s motivation to do the work in order to promote the physiotherapist’s goals. This duality is typical of the clown, which as aforementioned, is paradoxical in nature. The medical clown takes on the child’s limitations, resistance, and fear, while simultaneously holding the faith and hope that the child can make progress, improve, and devote themself to the process.

### 2.2. Amira

Five year old Amira underwent a medical procedure during which multisystem failure occurred. As a result of a lack of oxygen to the brain, she suffered irreversible brain damage and went from being a healthy child to one who needed to cope with a severe disability. Amira lost her ability to speak and lost control of her organs. In the first 2 months of her hospitalization, she suffered from restlessness accompanied by a lot of crying, involuntary limb movements, no control of her head, and hypertonia. Her condition at the time was characterized by drowsiness, and it was unclear whether she was responding to what was being said to her. The transition into this unfamiliar reality, which was accompanied by strange physical sensations, discomfort, and pain, while having no ability to express herself verbally as she once did, created great emotional distress. Amira was accompanied by her parents, who were also experiencing a deep rupture and loss following the traumatic event. The physiotherapy sessions were difficult for her and induced a lot of crying and uncontrollable emotional reactions. In light of this, the decision was made to have a medical clown join the treatments.

### 2.3. Data Collection and Analysis

The data include the treatment records written by the medical clown during Amira’s rehabilitation process and an interview with the physiotherapist who co-conducted the sessions with the medical clown. The analysis was designed to better understand the trajectory of the treatment and the role of the medical clown in its different phases [32]. First, the researchers (the two authors) read through all the treatment records. They then looked for data segments that represented phases in the process and coded them to identify chronological developments corresponding to the research questions [33]. They next reviewed the codes to define main themes in each phase that related to the role of the medical clown during each phase. Then, the first author presented the treatment records to the physiotherapist to obtain another perspective on the role of the medical clown. During the interview, the physiotherapist was asked to reflect on the treatment records in relation to two questions: (a) What was your experience during these sessions? (b) What was the role of the medical clown in Amira’s rehabilitation process? The next step was for the researchers to read through the interview transcript to identify data segments that represented themes in the process. The researchers reviewed and combined the themes from both data resources. These themes were defined and labeled, and selected quotes were chosen to illustrate each theme. Lastly, the findings section was structured chronologically to depict development in line with the research aim and questions [33].

## 3. Findings

The findings are presented as three phases in the process. Each phase captures one theme that relates to the role of the medical clown in the treatment process: (a) a playful space to enhance the inner motivation; (b) role expansion; (c) connection between the child and the environment.

### 3.1. The “Butterfly” Game: A Playful Space to Enhance the Inner Motivation

This theme relates to the ways the playful space provided by the medical clown enhanced the child’s inner motivation during the physiotherapy sessions. At the beginning of her rehabilitation process, Amira was restless and cried a lot during her physiotherapy sessions. Yael, the physiotherapist who was treating her, understood that, in order to conduct the treatments, she would have to create a safe and quite space for Amira. The goal of the physiotherapy at the time was to relax and lower Amira’s muscle tone, maintain her range of movement, and try to give her more control over her movements. Tsumi the medical clown began by making short visits to Amira’s room in the hospital, to allow them to get to know each other before she joined a physiotherapy session for the first time. When Tsumi entered the physiotherapy hall, Amira showed great interest. The physiotherapist introduced her and invited to her come closer to Amira with a calming voice. Amira looked at the clown directly, with a gaze that was inquisitive, wide awake, and full of vitality, fascinated by the clown’s red nose. This signaled to the clown that she could move even closer and advance the interaction. Amira looked at the two butterflies that were stuck in Tsumi’s hair and the physiotherapist said: “Do you see what she has on her head?” Amira’s mother noticed that the clown’s hat was actually a pair of underpants, and this made her laugh. She shared her discovery with Amira, which caused the child to laugh as well. At this point, it was clear that, despite her impairment, Amira understood humor, that she was intelligent and highly communicative, and that she could use facial expressions to convey interest or dissatisfaction, even if she could not express herself with words.

The clown took one of the butterflies out of her hair and moved it in the air as if it was fluttering. She sang a well-known children’s song about a fluttering butterfly in a soft, calming voice, as she let Amira follow it with her gaze. At this stage, Amira’s body language became calmer, her muscle tone had decreased, and she appeared fascinated by the song and the butterfly’s movement. The physiotherapist rocked her gently in her arms to the rhythm of the song and sang along. They sang the song together several times. The repetition of the pleasant sounds created a sense of safety and calm. After some time, the clown introduced another game. She put the butterfly on her shoulder as if it had landed there to rest, and then turned to it with an annoyed look. Amira was following this, waiting to see what would happen next. A moment later the clown sneezed, chasing the butterfly away, and Amira laughed with a big smile on her face. This game was repeated in several variations. Each time the butterfly landed on a different area (on the clown’s head, arm, or nose), it would bother her, and she would get rid of it until the next time it landed, sharing with Amira how annoyed she was with the butterfly. The clown continued to sing the pleasant song; however, as soon as the butterfly disturbed her, she would sing it in a scolding tone. Amira laughed and enjoyed watching the clown getting angrier and angrier each time. In the next stage, the clown put the butterfly on Amira’s hand as if it had landed there. The physiotherapist held Amira’s hand and helped her chase the butterfly away. In that moment Amira became more active and attempted an intentional movement for the first time since the therapy session had begun.

While reading the records of these sessions, Yael noted:

*I remember this period with Amira. She was very, very restless and it was very, very difficult to try to do anything, any kind of deliberate movement. I also remember this part with the butterfly… suddenly it became something like a game and her attention was elsewhere and it was calming and relaxed… it allowed us to see what she could express… and (it helps to) start trying to make a deliberate movement—to shoo the butterfly away… which we had not been able to do previously… This was a very important step, both for her and for those around her, because we could start to see what was there and what was not and that it was not so scary and that it would be fine. We could move forward from there…*

Amira was suffering from restlessness and involuntary body movements; thus, the clown chose to sing a slow and calming lullaby. Singing and following the movement of the butterfly that had come to life allowed Amira to enter into a dramatic reality, into a world of the imagination. She managed to relax and focus her attention, and the involuntary movements subsided. This allowed her to transition to the next stage—a game that emerged from the shared imaginary world that had been created in the room. This new game, involving the butterfly that kept landing on the clown, embodied a relationship between two roles: an active and playful one versus a passive and irritated one. Metaphorically, this reflected Amira’s physical and emotional state, as it dealt with the theme of wanting to rest versus the need to cope with restlessness and involuntary body movements that cause discomfort and exasperation. The irritating frantic butterfly was actually looking for a moment of peace; however, as soon as it stopped to rest, it made the clown restless. Thus, the butterfly represented the involuntary muscle movements acting on Amira. Amira could observe this dynamic as a bystander, from a safe distance, as the clown experienced the emotions of anger and discomfort while Amira watched and laughed, having a good time. Later on, when Amira chased the butterfly away, she became active. While she did not make the movement on her own, as Yael the physiotherapist made the movement for her, Amira was still able to play as if she were the one controlling the butterfly. This is a very simple game, but it allowed Amira to take on an active role and experience control, which was incredibly vital for her.

Yael commented on the role of the medical clown in enhancing the child’s inner motivation:

*This is the place I am looking for in therapy. When the movement comes from within the child and from her own drive and motivation… Clowning plays an important role… the clown enters the room and during the interactions with the child, and this helps the child find motivation and place and what interests her and what causes her to be in movement. This is a very important point in treatment. There are children for whom we cannot get to this stage without the help of the clown.*

Later, Yael talked about her own experiences dealing with Amira’s resistance to therapy at that time, and how the medical clown supported her in finding a way to stimulate Amira’s motivation:

*I remember there was enormous distress… because it was very difficult for me to deal with Amira’s current state. And there was a conflict, ok, I’m the physiotherapist and I have to do this and that. It is important to take care of her body, you have to take care of movement. But I quickly realized that there was no way we could work while she was crying and in distress. And you have to find another way. (At this point) I was also distressed and was looking for a way… And the clown… she was very important, she also helped me. The clown helps the therapist as well. It doesn’t only help the child.*

### 3.2. The Dwarves Game: Role Expansion

This theme relates to the ways the playful activities allowed role expansion and the development of the self. The next encounter took place about 8 months after Amira’s rehabilitation had begun. In this session, Amira was practicing a single intentional movement. The physiotherapist had been training her in different ways to move a hand or leg in an isolated, intentional, and controlled manner, and Amira’s abilities were improving. She practiced putting hoops on a cone, popping soap bubbles, doing yoga poses, and more. One of the games she played with the clown during treatments was a song-game called “Dwarf Dwarf in the Forest”. In this game, the child plays the dwarf, hiding curled up under a blanket or a piece of fabric and stretching out a hand or a leg according to the words of the song, until they come out entirely from under the cover and those present finally recognize who they are.

This structured game set the pace for Amira and gave her time to prepare herself before making each movement. She was met with great enthusiasm each time she managed to send out a hand or a leg from under the blanket. The clown, the physiotherapist, and Amira’s mother sang together for her, “Who is this dwarf who’s dressed up so beautifully?”, with all three of them acting curious to meet the dwarf and admiring his beauty. The dwarf song is a metaphor that deals with the theme of drawing within and hiding as opposed to being discovered and going out into the world. The exposure is gradual, giving the child a sense of control, as she is asked to move a different limb each time and knows what to expect.

Yael explained how the playful space suggested by the clown, such as in the dwarf game, supported the goals of Amira’s rehabilitation physiologically, as well as psychologically, by allowing her to re-explore the role of a child who engages in play like other children her age.

*During this period, we tried to work a little bit more on the control of movement, to achieve some kind of minimal control. So, for example the (dwarf) game really allowed her to… practice it over and over again… it’s impossible with a 5 year old girl to practice something boring. The game allowed her to enjoy practicing and really, really try hard and be intent (on her movements). Because that’s the meaning of this game… it was compatible with her age, compatible with her life and very suitable for the goals of the physio and she really liked it, and it was fun for her… it really brought back the feeling to her and maybe also to her mother that maybe it’s still possible to be a girl who does things that children do.*

This quote illustrates how this process allowed for self-continuity, since Amira was able to re-experience the playful activities as she engaged in before the traumatic event. The dwarf game also enabled transitioning from the inside to the outside world, which represents Amira’s way of coping psychologically with the loss she experienced. The moments of hiding reflect the need to go within and make the necessary preparations required for such a difficult physical task, which previously would have been so easy for her to perform. Here, Amira was able to explore emotions such as loneliness, uncertainty, and fear of encountering the world. Coming out from under the blanket can be seen as a metaphor for Amira’s growth, a rediscovery of her capabilities, and her ability to improve them, as if saying “I can move and play in my new condition”. This is how Tsumi the clown described the game in her treatment diary: “When Amira came out from under the fabric completely, we were full of joy and admiration. I saw the light and excitement in her eyes, and thought to myself how important and meaningful it was that her mother was present there with us, that she had a chance to enjoy and celebrate her love for Amira”. The game allowed Amira to experience the fact that she was loved, that she inspired enthusiasm, and that it was fun and enjoyable to be and play with her, all of which allowed her to develop her ability to adapt and accept her new condition.

In a sense, the game echoed Amira’s physical and emotional state in the first stages of her hospitalization. It depicts the process of transitioning from the inner to the outer world, from unconsciousness to awakening and connecting with her surroundings. In light of the new disability to which Amira and those around her had to adapt, the game can also evoke the association of rebirth. The words of the song in the dwarf game, “Who is this dwarf?”, contain a question pertaining to identity. This question is highly relevant to a child when one of their greatest tasks is becoming reacquainted with herself and allowing those around to become reacquainted with them. There is an expectation and perhaps even concern regarding what will happen when they encounter the outside world—how will they be received? Step by step, Amira is invited to come out into the outer reality in an organized and gradual manner.

Later on, it was Tsumi’s turn to hide under the blanket, while Amira told her which body part to send out each time. Now, Amira was controlling the game. She could look at the clown, who, in a sense, was mirroring her own story, as she played the part of the audience and even directed and led the situation. The clown often got tangled up and did silly things, in an aim to heighten Amira’s success and sense of competence.

At this stage of the game, Amira was experiencing a process of role reversal. No longer was she in the role of being passive and having no control; now, she was playing the role of a leader who could guide, control, choose, be mischievous and playful, and have fun as she used to before the trauma she had experienced. From this position, she was able to experience a sense of competence and control she so needed, while the shift between roles allowed her to explore various states of self.

Yael added her comments on the dwarf game, and how the interaction with the clown enriched the game and allowed for role expansion:

*I think that when the clown was there, the game was richer. I could play the dwarf game with her when the clown was not around. But once the clown was there, the focus was on the game… there was much more laughter and much more joy and much more movement. And then there is the positioning, which is very important, when they change roles, she tells the clown what to do… like the kids in kindergarten take turns, which doesn’t usually happen in normal physiotherapy. So, she (Amira) could also be in the leader role not only the follower… The game became very complete.*

### 3.3. The Shop Game: Connections between the Child and the Environment

This theme relates to the role of the clown in creating connections between the child and the environment which was more visible during the last phase of the rehabilitation process. Toward the end of the therapeutic process, about a year and a half since Amira’s rehabilitation had begun, she seemed to be experiencing greater acceptance and an ability to cope with her disability, alongside a sense of competence. She liked the familiar routine of treatment activities, she felt loved and protected by the staff and her family, and she was enjoying her ability to move. She could communicate, choose, laugh, and play. She did not regain the ability to speak; however, she did learn to communicate “yes” and “no” by making a sound and moving her mouth and with facial expressions. She had been practicing walking with different aids and was now using a special walker that enabled assisted mobility for short distances. Each step she made was met with praise and admiration, and she was working hard and getting stronger. This was the final period of her rehabilitation, and she was preparing to go home and back to school. The physiotherapy sessions were devoted to working on strengthening and controlling her body and working on mobility. During the sessions, she would leave the physiotherapy room to practice walking in the hospital hallways.

One of the games Amira played with the physiotherapist and the clown during this period was the “shop” game. At the start of the session, while the physiotherapist was helping Amira stretch on the mattress, Amira and Tsumi played with sticking colorful balls onto sticks. Tsumi attached a ball to a stick and said to Amira, “Look! It’s a lollypop! You want one too?” Amira said yes, and they made quite an impressive collection of lollypops, tasting them and sharing them with Amira’s mother and the physiotherapist. They all ate the lollypops with delight. Yael the physiotherapist suggested they sell them outside the physiotherapy room, with the intention of training Amira to use the walker. Amira happily embraced the idea, and Tsumi enthusiastically added that they should go to the hospital cafeteria and sell them there. Yael sat Amira on the walker and fastened the straps and Amira began to walk out of the physiotherapy hall. At the entrance to the hallway, Tsumi blew an imaginary trumpet and announced Amira’s arrival in a grand voice, as passersby smiled and took interest. “Dear audience, give it up for the one and only, Amira the sweetie-pie and her sweet lollypops! Now on sale! You taste—you pay! You wanna taste? These are very special lollypops that Amira made with her own two hands”. They invited everyone who was there to buy a green pickle-flavored lollypop or a purple fried eggplant-flavored lollypop. Staff members, parents, and children showed interest and surprise, responding with a sense of humor when they heard about the bizarre flavors and outrageous prices. Step after step, happy and laughing, Amira progressed toward the cafeteria—the most central place in the hospital, where multiple interactions take place with a variety of people.

Choosing to leave the physiotherapy room and walk toward the cafeteria is indicative of a desire to explore possibilities and experience social belonging. It allowed Amira to play a central and active social role and experience herself as being able to give to others by “selling” the pretend lollypops she had made. Tsumi the medical clown encouraged uninhibited playfulness, connection, and interaction, thereby inviting Amira to playfully explore her environment.

Amira, who had experienced such a devastating loss, played a role that was full of confidence, the role of one who lacked nothing and, on the contrary, had something to give to those around her. She looked at people directly, feeling proud of herself, initiating contact with the world, and inviting others to take part in her goodness and sweetness. Amira, who was once completely helpless, was now regaining the control she had lost. She was learning to accept her disability, playing and enjoying what she did have, and feeling worthwhile as she experienced abundance and belonging.

Yael commented on the ways the medical clown supports the connection between the child and the environment:

*The clown makes all these connections, you know, connects it to life, to others, to the outside… Now that I think about it, she couldn’t speak, she couldn’t say that she was coming… She could play… with us, but she couldn’t tell people: “come by, I made candy!” So, the clown was her voice. And this is an important role. She suddenly plays with everyone in the hallway, and everyone obeys her, because the clown is walking beside her and saying what she cannot say… thanks to the clown the environment was more appropriate and enabling.*

Finally, Yael also reflected upon what she learned from the medical clowns she worked with:

*I learned to be a bit of a clown myself and to use… a bit of these qualities… to take a step back, to give children their place, the stage, to let the children reveal themselves. I learned to work calmly and not out of need… the clowns allow all this, and I learned from them. Because the clowns are not always there. I wish there was always a clown in (physio)therapy.*

## 4. Discussion

Accidents and serious injuries are defined as life-changing traumatic events, especially when they involve a physical impairment that affects functioning [26]. Amira, once a healthy child, experienced severe trauma following complications that occurred during a medical procedure that almost cost her life and forced her to have to cope with an extreme disability. There is no doubt that Amira and her family experienced a major loss following the event, which required them to restore and restructure every aspect of Amira’s life [34,35].

Physiotherapy treatments are a very significant part of the process of restoring functions and restructuring daily life after experiencing loss. These treatments build and enhance patients’ physical capabilities and emotional resources [36]. When children are resistant to treatment, involving a medical clown in the process can help motivate them to cooperate. The qualitative analysis shows how the medical clown’s ability to establish a playful space in the rehabilitation process enhanced the child’s inner motivation, provided a space for role expansion, and promoted the connection between the child and the environment.

When Tsumi the medical clown joined Amira’s physiotherapy sessions, she introduced a playful dimension that helped Amira progress toward the physical goals of the treatment. At the same time, this allowed her to deal with psychological content that was relevant to processing the loss she had experienced and helped her adapt to her new condition. The clown’s participation made it possible to simultaneously focus on the two coping strategies referred to above [29,30]: the loss-oriented strategy of expressing and processing difficult feelings entailed in the loss, such as sorrow, pain, fear, and loss of control, and the restoration-oriented strategy of helping Amira organize and relearn her abilities, form new relationships, and rebuild her positive identity.

Theories that deal with processing loss and structuring meaning around situations of bereavement highlight the need to build an enveloping, containing environment that allows the individual to feel secure. As Neimeyer and Rynearson (2022) noted, in order to conduct a restorative work of the loss story in therapy, there is a need to establish a trusting and collaborative therapeutic relationship [37]. The various stages of Amira’s therapeutic process describe play processes based on well-known rituals and songs as a means for calming her and strengthening her sense of security. The repetitive songs and games in which various situations were repeated allowed her to safely and gradually enter a playful dramatic space and explore various aspects of loss and growth.

The dramatic work in the butterfly and dwarf games enhanced her inner motivation and allowed Amira to shift between roles that had qualities of control and competence and roles that lacked control and were chaotic and passive. The games depict the dynamic between the roles created through aesthetic distancing [17,18]. The passive role played by the clown is in fact a reincarnation of the role of “patient” [25]. This role is characterized by the fact that the hospitalized person’s privacy and control of their body is violated as a result of their injury and being hospitalized. It contains unexpressed emotions, thoughts, and experiences such as anger, helplessness in the face of feeling invaded, restlessness, and frustration. The situation played out in the games is a metaphoric reflection of the inner experience. However, in the dramatic reality, the medical clown reflected the situation for Amira in a way that was playful, spontaneous, and full of humor, by using aesthetic distancing: “It’s the butterfly that’s bothering me”; “It’s the clown who got tangled up in the blanket and can’t get out”. This allowed Amira to encounter and explore the difficult emotions she was experiencing in her life.

Alongside roles that lack control, which represent the losses caused by the impairment, the games allow other roles to be expressed—roles that are active, in control, and competent. For example, in the butterfly game, when the butterfly bothered Amira, it made her laugh and she was able to use those around her to get rid of it, as the butterfly was the one who was helpless. Thus, the dramatic game allowed Amira to expand her repertoire of roles [20] and express qualities of self that where active and able to experience pleasure, control, and strength. In this way, Amira could explore situations that had previously been inaccessible to her since her injury, while training her body to return to a state in which she was controlling it and not the other way round.

Playing with the opposing roles of passive and active through the various games created an experience of integration. Much like muscle training, the repetitive shifts between the opposing stances helped to practice narrowing the gap between anxiety and calmness, between helplessness and competence, and between the inner and the outer. The clown helps achieve this mission because she herself is a flexible character, full of contradictions, and able to constantly shift between opposites [2,22]. The goal of drama therapy is to develop a balanced, flexible, and healthy role system and expand the range of roles available to us, while helping integrate conflicting roles and accept the ones we deem less legitimate [20,24]. The clown can be viewed as a character that shifts between the conflicting roles, holding and exploring them and, thus, allowing them to become integrated.

In addition, the repetitive shifts between the opposing positions in the various games are similar to the oscillation described in Stroebe and Schut’s (1999) adaptive model of coping with bereavement [29,31]. The oscillation between processing the sorrow and difficulty and focusing on restoration and taking action takes place within a game, as the clown sets the pendulum in motion and helps the child shift between the strategies while the physiotherapist holds the process of restoration and regrowth.

Successful rehabilitation processes are based on the individual’s perception of self-efficacy, which stems from the way the environment perceives their disability and their own self-image, body-image, and mood [27]. Toward the end of her rehabilitation process, Amira had made peace with her new condition; she could accept it, could cope with it, and was able to communicate with the environment. In the “shop” game, Amira was happy and active as she confidently went out into the hospital space, knowing that she could contribute to the other children and parents at the hospital. She could initiate contact with them and draw them to her out of a sense of joy and self-worth. Amira experienced independence as she walked using her special walker, which supported her physically.

It is possible to see how personal content that was brought into and explored in the dramatic reality was transformed. Content that was difficult and oppressive was brought into a safe and playful space and was embodied in a way that was tangible, concrete, and alive, allowing for new perspectives of the loss story to be discovered [37]. The possibility of entering the reality of a game and acting within it provided the opportunity to be creative and active, as in an act of creation.

While this work discusses a particular case, it is undoubtedly representative of many others. The observation presented here contributes to our understanding of the role that medical clowns play in children’s rehabilitation processes. Much like Amira, many children in rehabilitation experience a profound sense of loss and helplessness following traumatic events. These children need to rediscover their strengths, regain a sense of control and self-efficacy, and restore their self-worth. However, one limitation of this case study is that it is based on the medical clown’s diary and the physiotherapist’s point of view, but it does not include the child’s or her parents’ perspectives. Future studies should integrate information from diverse sources (such as the child, parents, and medical staff) to gain an in-depth understanding of the phenomenon. In addition, future studies that make use of additional research tools such as quantitative measurements or qualitative interviews would provide a broader perspective of the influence the processes described here have on psychological and physiological health indicators.

## 5. Conclusions

The primary contribution of the current work is the opportunity it provides to gain an in-depth understanding of the role of medical clowns in children’s rehabilitation. It sheds light on the purpose of their red nose, their unique presence, and what they do as part of the rehabilitation process. An acquired disability such as Amira’s requires coping with a substantial loss of functioning. Many children in rehabilitation face a similar task of having to adapt to a new reality and find the strength to cope with fear, pain, and emotional stress. Involving medical clowns in rehabilitation processes can help children surmount this essential and complex process.

Medical clowns invite these children into the playful space of a dramatic reality where they can process complex and difficult emotions, as the clowns use aesthetic distancing to approach this content. The current work demonstrates how the playful encounters with a medical clown provide a meaningful psychological exploration of feelings of helplessness and lack of control in the face of major losses, while helping the children access their ability to cope and grow from their new condition. The clown brings with her a flexible spirit, helping the children practice inner flexibility and move between various states of self, thereby allowing them to expand the repertoire of roles available to them. Children can transition from the role of the passive “patient” who has no control to roles that are strong, in order to enhance their sense of security and trust in the treatment and in themselves. This allows them to process the loss, create meaning in their new condition, and significantly promote their rehabilitation processes.

## Data Availability

The data presented in this study are not available due to restrictions of privacy.
